# Pharmacology education for nurse prescribing students – a lesson in reusable learning objects

**DOI:** 10.1186/1472-6955-7-2

**Published:** 2008-01-23

**Authors:** Joanne S Lymn, Fiona Bath-Hextall, Heather J Wharrad

**Affiliations:** 1 School of Nursing, University of Nottingham, Queens Medical Centre, Nottingham, UK

## Abstract

**Background:**

The shift away from a biological science to a social science model of nursing care has resulted in a reduction in pharmacology knowledge and understanding in pre-registration nursing students. This has a significant impact on nurse prescribing training where pharmacology is a critical component of the course from a patient safety perspective.

**Methods:**

Reusable learning objects (RLOs) are electronic resources based on a single learning objective which use high quality graphics and audio to help engagement with the material and to facilitate learning. This study used questionnaire data from three successive cohorts of nurse prescribing students (n = 84) to evaluate the use of RLOs focussed around pharmacology concepts to promote the understanding of these concepts in students. A small number of students (n = 10) were followed up by telephone interview one year after qualification to gain further insight into students' perceptions of the value of RLOs as an educational tool.

**Results:**

Students' perceptions of their own understanding of pharmacology concepts increased substantially following the introduction of RLOs to supplement the pharmacology component of the course. Student evaluation of the RLOs themselves was extremely positive with a number of students continuing to access these tools post-qualification.

**Conclusion:**

The use of RLOs to support the pharmacology component of nurse prescribing courses successfully resulted in a perceived increase in pharmacology understanding, with some students directly implicating these educational tools in developing confidence in their own prescribing abilities.

## Background

As part of the government's commitment to a more modern National Health Service (NHS) which allows patients quicker and more efficient access to medicines, prescribing powers have been extended to include not just doctors and dentists but also nurses who have undertaken a post-registration prescribing qualification [1]. Over the past few years we have seen both the implementation of supplementary prescribing for nurses [2] and the gradual expansion of the independent formulary for nurses [3,4] such that nurse prescribers are, in effect, able to prescribe the same drugs as doctors. The implications for patient safety following this expansion of nurse prescribing rights clearly signals the need for robust teaching strategies to promote understanding of new concepts and development of competency.

While the prescribing rights of qualified nurse prescribers in the UK are not dissimilar to those of advanced nurse practitioners with prescriptive authority in both Australia and USA there are some considerable differences in how these nurses are educated to perform this role. Currently the requirements for undertaking a prescribing course in the UK are that nurses should be able to study at degree level and must have been qualified for a minimum of 3 years [5]. In the UK there is however no requirement for nurses to show that they have previously studied at degree level nor is there any requirement for them to demonstrate a background level of understanding of basic biological sciences, a concern that has been previously raised by Tyler and Hicks [6]. This contrasts with the USA and Australia where, following research into nurse practitioner education, these programmes are taught at master's level as a minimum [7,8].

With the shift in pre-registration nurse training away from a medical model to a social model of care the level of biological science knowledge and understanding gained by pre-registration nurses has significantly reduced [9]. Indeed a number of studies have identified pharmacology education as an area of significant weakness both in the UK and elsewhere [10-12]. The implications of this for nurse prescribing education are profound [13] with a number of studies suggesting that both nurse prescribing students and educators recognise the problems associated with providing an adequate level of pharmacology input into these courses. [14,15]

While pharmacology is only one of eight areas of indicative content for nurse prescribing courses (Table 1) it impacts on the majority of the remaining areas of indicative content and as such is important to ensure the safety of nurse prescribing, as it is with medical prescribing [16]. Pharmacology knowledge is examined as a separate assessment, by means of multiple choice and short answer questions, at the end of the nurse prescribing course and there is no compensation across assessments so students need to pass this exam to obtain the professional qualification. In order to promote safe prescribing we should be encouraging nurses not just to memorise enough pharmacology to pass the exam but to assimilate this knowledge and be able to integrate it (both vertically and horizontally) into their clinical practice. In this sense vertical integration can be defined as the integration of fundamental pharmacology principles into clinical therapeutics while horizontal integration refers to the integration of these principles into a comprehensive and holistic view of patient care [17].

**Table 1:** Indicative Content for Nurse Prescribing Courses

1	Consultation, decision-making and therapy including referral.
2	Influences on and psychology of prescribing.
3	Prescribing in a team context.
4	Clinical pharmacology including the effects of co-morbidity.
5	Evidence-based practice and clinical governance in relation to nurse prescribing.
6	Legal, policy and ethical aspects.
7	Professional accountability and responsibility.
8	Prescribing in the public health context.

This table shows the indicative content for nurse prescribing courses as detailed by the Nursing & Midwifery Council [5]

Unfortunately teaching basic pharmacological concepts is a dry business and many students find it difficult to relate these concepts to clinical experience. Teaching these types of concepts is made more complex by the different academic levels and capabilities of nurses accessing nurse prescribing courses [14]. If we include the lack of classroom time available for nurse prescribing courses (only 26 days) coupled with the variety of clinical backgrounds and expertise of nurses attending these courses we can see that developing pharmacological understanding in students is a difficult task. Indeed in the study by Bradley and colleagues a number of lecturers involved in nurse prescribing training commented that their students had particular concerns with pharmacology and would benefit from preparatory material covering basic principles to develop familiarity with pharmacological terminology [14].

Perhaps part of the problem lies with the nature of pharmacology education for nurses which appears to utilise traditional teaching methodologies almost exclusively [15]. While lectures result in delivery of information they do not necessarily engender learning and understanding which may be better supported by blended or more applied teaching methodologies.

Consequently in order to promote the required understanding of pharmacology concepts, and taking into account the developments in e-learning technology, we have developed a number of flexible learning tools known as reusable learning objects (RLOs). Reusable learning objects (RLOs) are discrete units of learning that can be integrated into a formal lecture or used individually to aid revision or background knowledge. These learning objects use high quality graphics and audio to help engagement with the material and to facilitate learning. The concept of RLOs has already been identified as an important issue for new medical curricula abroad [18,19] and they have even been identified as improving learning performance of students studying clinical laboratory sciences [20].

Each of our reusable learning objects deals with a single pharmacological concept, is designed to take no more than 30 minutes to work through, and includes short interactive assessments to test developing knowledge. The subject of each RLO was specifically chosen as it represents a fundamental principle, an understanding of which is important not just for the pharmacology assessment but also as a building block for clinical therapeutics. These RLOs are freely available through the School of Nursing website and can therefore be accessed by students from home or work as well as from within the University, thus allowing students' greater flexibility in their learning (All the RLOs used in this project are open access with their web addresses being detailed in Table 2). This flexibility is particularly important for post-registration nursing students who may have both work and family responsibilities in addition to being a student. Indeed web-based open and flexible learning modules have previously been used with some success for post-registration nurse education including prescribing [21].

**Table 2:** Pharmacology RLOs made available to Nurse prescribing students.

RLO Number	RLO Title	Cohort(s) Available to	Web address for RLO
1	Exploring the synapse	Cohort 2 Cohort 3	
2	Bioavailability	Cohort 2 Cohort 3	
3	Half-life of drugs	Cohort 2 Cohort 3	
4	Understanding First Pass Metabolism	Cohort 2 Cohort 3	
5	Lock and Key hypothesis	Cohort 3	
6	Plasma proteins and drug distribution	Cohort 3	
7	The kidneys and drug excretion	Cohort 3	
8	The liver and drug metabolism.	Cohort 3	

This table indicates the pharmacology RLOs developed and which cohorts of students these were available to along with web addresses for these RLOs. These RLOs are free to use for educational purposes under the Creative Commons Licence. The authors would be grateful if users would complete the short online evaluation forms at the end of the RLOs to help with our continued research studies.

This paper describes the evaluation of these pharmacology RLOs and their value in promoting the understanding of pharmacological concepts between different cohorts of nurse prescribing students at the University of Nottingham between January 2004 and September 2005.

## Methods

All students entering the nurse prescribing course at Nottingham University between January 2004 and September 2005 were invited to take part in this study. The study protocol was explained to all students and all students were given the opportunity to opt out of the study. Participation or lack of participation did not impact on the way in which students were taught and did not influence progress on the course.

As an evaluation of the value of an educational tool conducted mainly within class time ethical approval from the University research committee was not required. Experimental design and analysis was however performed following the British Educational Research Association's revised ethical guidelines (2004) and all data were anonymised before publication.

RLOs were developed using the Universities Collaboration in Elearning (UCel) framework [22] and comprised a multistage process including expert review at two different stages of development and formative student evaluation prior to use with student groups.

**Cohort 1 **(34 students) – This cohort of students followed the standard course module with pharmacology being delivered by didactic lecture format. Students were provided with book lists, weblinks and some general CAL materials but no RLOs were provided to supplement the pharmacology teaching.

**Cohort 2 **(36 students) – This cohort of students followed the same course and had access to the same resources as Cohort 1 but with the addition of RLOs 1–4 (Table 2) that could be accessed by the student at any time in the course. RLOs were not used in lectures but were introduced to students in a separate session. Students were also advised on the timetable about RLOs which might be suitable supportive learning tools for specific lectures.

**Cohort 3 **(14 students) – This cohort of students again followed the same course but had access to all eight pharmacology RLOs (Table 2) and introductory RLOs on the anatomy and physiology of both the liver and kidney.

Questionnaires were distributed to students at the end of the pharmacology component of the course and students who did not attend class on that occasion were asked to complete the questionnaire at the following session.

### Value of RLOs in promoting pharmacology understanding

This study involved the collection of questionnaire data from three successive cohorts of nurse prescribing students regarding their perception of their own understanding of pharmacological concepts and whether they have been able to apply this to clinical practice. This questionnaire consisted of 26 items and was a mixture of both fixed response and open response questions. Students rated their perceived understanding of 10 specific pharmacological concepts (including, but not exclusively, concepts represented by RLOs) on a five point scale ranging from 'very badly' to 'very well'. Students were also asked to rate their own confidence in understanding pharmacology on a four point scale ranging from 'very confident' to 'not confident'. The questionnaire also contained 3 open response text boxes asking 'What was the most valuable resource that you used to clarify your understanding of pharmacological processes', 'In relation to your nurse prescribing practice please comment on any elements of the pharmacology component you found to be particularly beneficial' and 'Please give a specific example where you have used the knowledge of pharmacology you acquired during the course in your nurse prescribing practice'. Cohorts of students who had access to RLOs were also asked to rate the value of the RLOs for pharmacology teaching against other resources such as course notes and textbooks with 1 being the most useful and 6 being the least useful.

The questionnaire was designed following discussion between the authors and was checked for content validity by the module leader for the nurse prescribing course. Following distribution to cohort 1 no issues regarding face validity arose so there were no subsequent changes in the questionnaire for cohorts 2 and 3.

### Evaluation of individual RLOs

Cohorts 2 and 3 were also asked to evaluate the RLOs using a questionnaire which comprised 29 fixed response questions using a 6 point scale using the descriptors 'Not applicable', 'Strongly agree' 'agree', 'neutral', 'disagree' and 'strongly disagree'. The questions were arranged into sections concerning access to computers, usability, look and feel, content and reuse. The questionnaire also included 3 open response text boxes asking 'What did you like about this RLO?', 'How could the RLO be improved?' and 'Any further comments?'

Students were introduced to the concept of RLOs and provided with evaluation forms for individual RLOs in a computer lab session. This ensured that all students were aware of the available RLOs and where to find them. An information technologist was on hand in this session to deal with technical and navigational problems issues which arose through using the university system. There was no pharmacological input from a lecturer in this session. All evaluation forms were completed anonymously and placed into a box by students at the end of the session

As these RLOs were freely accessible from the School of Nursing website these evaluation questionnaires were also completed by other students, such as those studying for a Masters in Nursing Science, who had accessed them.

### Impact of RLOs for pharmacology on Clinical practice

An independent researcher was employed specifically to conduct telephone interviews with students one year after successful completion of the nurse prescribing course in order to gain an insight into the impact of the pharmacology RLOs on their continuing clinical practice. The independent researcher randomly selected students from among those who had attended the course as part of cohorts 2 and 3 for interview by telephone. 10 students were interviewed representing 15% of the total students from these cohorts, were interviewed by telephone. The interview was semi-structured and was piloted on two prescribers before being utilised in this study. Student responses to the questions shown in Table 3 were noted down in written form and a summary table of responses was compiled.

**Table 3:** Students perceptions of the impact on their practice of RLO learning in pharmacology.

Question 1	Do you recall/Did you use the RLOs that supported the course/Did you have any problems accessing them?
Question 2	What properties of the RLOs helped your learning? (prompt media components. Verbal, visual, diagrams, animations .....)
Question 3	Have you used the RLOs again? (when, how often ...?)
Question 4	Have they had any impact on your practice?
Question 5	Do you see any value in this method of teaching pharmacology?
Question 6	Have you used other RLOs?
Question 7	Have you recommended the RLOs to others?
Question 8	Would you like to see more developed? What topics would you like to see developed as RLOs?
Question 9	Do you have any suggestions for improvements of the RLOs
Question 10	Do you feel confident in your understanding of the pharmacology of the drugs you prescribe?
Question 11	What is your highest biological science qualification?

Questions explored in the telephone interviews of nurse prescribing students one year after completing the course.

This researcher had had no previous involvement in the nurse prescribing course, was not known to any of the interviewees and is not an author of this paper.

## Results and Discussion

### Educational background of nurse prescribing students

**Figure 1:**
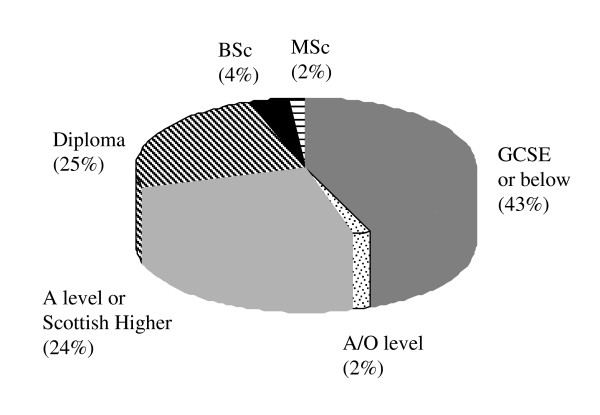
**Highest Biological Science Qualifications**. Highest Biological Science qualifications of students on Nurse Prescribing Course. Combined data from September 03 and January 04 cohorts (70 students).

Of the nurse prescribing students entering the course at the University of Nottingham (n = 100) 95% were female and had had a mean of 20.8 ± 0.7 years of nursing experience, ranging from 4 years through to 34 years. These students also exhibited a huge variation in formal biological science qualifications ranging from none all the way up to Master's level. The wide range in academic ability within cohorts has been previously noted by nurse prescribing lecturers in the West Midlands [14] and according to a recent study by Courtenay et al is reflected nationally [23]. Perhaps the most important difference between our data and that of previous studies is that we have specifically obtained students' highest biological science qualification rather than their highest academic qualification, which for many nurses is social science based. It can be seen from our data that almost half of students attending the nurse prescribing course had no more than a GCSE in a biological science subject (Figure 1). While 25% of students stated that they had a diploma in a biological science a number of students classified their nursing diploma in this category, the shift in the pre-registration nursing curriculum away from a biological science model suggests that the biological science component of these courses may be limited. Overall then the majority of students had little in the way of formal biological science qualifications making the delivery of what should be degree level pharmacology to these students a very challenging process.

While 100% of students from cohorts 1 and 2 completed the study only 47% of students from cohort 3 completed the study although this was likely the result of timing, with final questionnaires being handed out on the last taught day of the course. While a number of students took the questionnaire home with them to complete these were not returned to the University for inclusion in the analysis.

### Evaluation of pharmacology RLOs

Student feedback indicated that students spent between 8 and 15 minutes working through each RLO with 'First Pass Metabolism' taking the shortest time to work through (8 ± 3 minutes) while 'Half Life' took the longest time to work through (15 ± 6 minutes). In terms of the usability and media attributes of the RLOs the majority of students either agreed or strongly agreed with statements relating to the ease of use of the RLOs and the value of the different components which make up the RLO, although 25% of students did comment that the RLO took longer than expected to complete (Tables 4 and 5).

**Table 4:** Student ratings of usability of RLOs.

	Strongly Agree	Agree	Neutral	Disagree	Strongly disagree	N/A or missing
The RLO was easy to use	39%	53%	4%	2%	0%	2%
The RLO was easy to navigate. I felt in control	42%	51%	4%	2%	0%	2%
The RLO was well structured and easy to follow	47%	47%	4%	1%	0%	1%
I liked the look and feel of the RLO	34%	47%	14%	3%	1%	2%

Percentage of students (cohort 2 and cohort 3) rating the pharmacology RLOs in terms of statements relating to usability.

**Table 5:** Student ratings of media attributes and size of RLOs.

	Strongly Agree	Agree	Neutral	Disagree	Strongly disagree	N/A or missing
The images and animations were valuable components of the RLO	48%	45%	5%	1%	0%	1%
The on-screen text was useful and helped me assess the amount of information each section contained.	28%	54%	9%	1%	0%	9%
The RLO took longer to complete than expected	6%	19%	23%	37%	15%	0%
The narration made the RLO more engaging. I preferred this to text alone	32%	44%	7%	7%	0%	14%

Percentage of students (cohort 2 and cohort 3) rating the pharmacology RLOs in terms of statements relating to their media attributes and size

With respect to educational value and learning support students were overwhelmingly positive about the value of RLOs with 90% agreeing or strongly agreeing with the statement 'The RLO has aided my understanding and I feel I have achieved the learning objective' and 92% agreeing or strongly agreeing with the statement 'I am confident that I will be able to use the knowledge gained from this RLO in future practice' (Table 6). Comments made in the open questions backed up this positive view of RLOs;

**Table 6:** Student ratings of educational value and learning support of RLOs.

	Strongly Agree	Agree	Neutral	Disagree	Strongly disagree	N/A or missing
I needed the help of a lecturer to understand the content	5%	22%	15%	28%	26%	5%
The RLO was interesting and engaging	38%	53%	5%	2%	0%	2%
The RLO was pitched at the right level for me	26%	50%	14%	5%	4%	1%
I needed more support when using the RLO	4%	24%	15%	31%	23%	4%
The content was appropriate and fitted my learning needs	26%	64%	7%	2%	0%	1%
The activity was appropriate and aided my understanding	30%	61%	6%	3%	0%	1%
The RLO encouraged me to reflect on the material	23%	62%	10%	2%	1%	2%
I am confident that I will be able to use the knowledge gained from this RLO in future practice	34%	58%	7%	2%	0%	0%
The self-assessment helped me gauge how well I'd understood the material	37%	47%	7%	4%	1%	4%
The RLO has aided my understanding and I feel I have achieved the learning objective.	35%	55%	7%	3%	0%	0%
The RLO will help me retain information	39%	45%	11%	2%	1%	3%
I think it's useful to supplement lectures with RLOs like this one	47%	38%	4%	2%	0%	8%
I will use this RLO again	37%	45%	4%	2%	2%	9%
The RLO integrated well with the module and other teaching sessions	29%	56%	4%	2%	0%	9%

Percentage of students (cohort 2 and cohort 3) rating the pharmacology RLOs in terms of statements relating to their educational and learning support value.

'Liked the visual analogues, fantastic way of learning and remembering'

'Very practical in terms of use/understanding in practice. Excellent learning tool'

'It is a useful tool for revision, particularly being able to pace it and return to material to verify understanding'

There were some negative comments but these were based almost exclusively around issues of access;

*'Would like to use the computer assisted, thought session was informative – but didn't know how to access from home computer'*.

Previous investigations of the effectiveness of e-learning technologies for health professionals identified a number of barriers to its success among them cost, poorly designed packages, lack of skills, need for a component of face-to-face teaching, time intensive nature of e-learning and computer anxiety [21,24]. The value of these pharmacology RLOs may be partly because they are by their very nature different to traditional e-learning tools. Our data suggest that individual RLOs do not require more than around 15 minutes to complete thus they do not require a time-intensive input making them more flexible for students to use at work or home. Similarly the visual, audio and interactive nature of these RLOs means that they have an appeal for visual, auditory and kinesthetic learners an important issue bearing in mind data which suggests that learning style is important in web-based e-learning [25,26].

### Learners' Perceptions of Pharmacology Understanding

**Figure 2:**
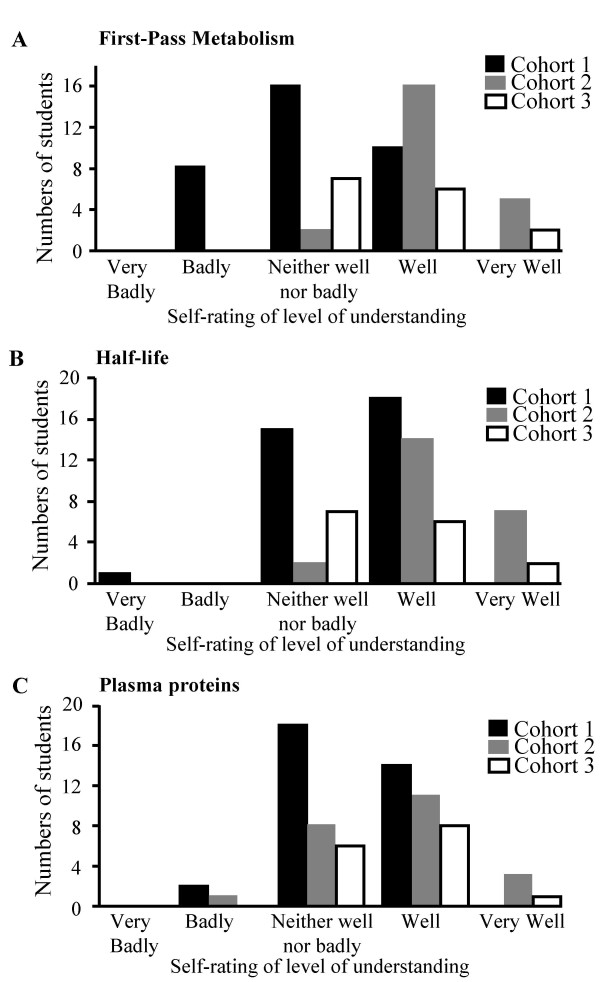
**Perceived understanding of pharmacology concepts from 3 cohorts of students**. Students from three consecutive cohorts of the nurse prescribing course were asked to rate their understanding of pharmacology concepts on a five point scale (very well to very badly), on completion of the course. The figure shows the trends for 3 concepts. a) Understanding of first pass metabolism. b) Half-life of drugs. c) Plasma proteins and drug distribution. Cohort1 did not have access to any RLOs; cohort 2 had access to RLOs 'Understanding First Pass Metabolism' and 'Half Life of Drugs' and cohort 3 had access to all three RLOs. When RLOs were available the distributions shifted to the right away from the Badly/Very Badly descriptors to the Well/Very Well pole. The shifts were significant (Kruskal Wallis p < 0.05) for 'Understanding First Pass Metabolism' and 'Half Life of Drugs'.

The overall distribution of students' perception of their understanding of pharmacological concepts shifted to the right (towards the 'well' and 'very well' pole) for cohorts 2 and 3 who had had access to RLOs. Indeed when RLOs supported the concept no student rated their understanding as 'bad' or 'very bad'. Statistical analysis comparing the responses of the three cohorts was carried out using a Kruskal Wallis test and demonstrated a significant difference between mean ranks of the 3 cohorts in all but 2 RLOs and these data including 'p' values are shown in Table 7. The use of these two RLOs did suggest an increase in student understanding of the pharmacological understanding of the concept. However these RLOs were only available to cohort 3 which had the lowest number of student responders and it may be that the lack of statistical significance is a direct result of this reduced 'n' number. Histograms of the change in students' perceptions of three separate pharmacology concepts are shown in Figure 2.

**Table 7:** Comparison of ratings of understanding of pharmacology concepts.

RLO Number	RLO Title	Chi Square	'p' value
1	Exploring the synapse	8.6	0.014
2	Bioavailability	16.5	0.001
3	Half-life of drugs	15.1	0.001
4	Understanding First Pass Metabolism	24.2	0.001
5	Lock and Key hypothesis	5.9	0.053
6	Plasma proteins and drug distribution	3.9	0.142
7	The kidneys and drug excretion	13.7	0.001
8	The liver and drug metabolism.	10.1	0.007

Kruskal Wallis test to compare the ratings of perceived understanding of each pharmacology concept between the 3 cohorts of students (2 degrees of freedom).

As can be seen from Table 8 students from cohorts 2 and 3 ranked RLOs third behind lecture notes and text books in terms of the learning resources which they felt had supported their learning best throughout the course. The RLOs were however rated substantially higher than journals, CAL packages and other web resources.

**Table 8:** Ranking of RLOs against other learning resources.

Learning Resource	Average Rank
Lecture notes	1.4
Text books	1.8
RLOs	2.9
Journals	4.1
Web resources	4.6
CAL	5.2

Students ranking of RLOs against other resources to support pharmacology learning (n = 11 students)

It should be remembered that these RLOs were used within the nurse prescribing course to support and supplement, rather than replace, face-to-face teaching. This ensured that students maintained a relationship with a pharmacology lecturer who they saw on a regular basis and with whom they could discuss issues arising out of either lectures or RLO use. The ability to access the RLOs any number of times was also important, one student who had difficulty understanding what a synapse was said about the 'Exploring the Synapse' RLO:

"This one was good to go over and over again until I got it right and for revision"

The main problem associated with RLO use was focussed around computer anxiety and accessibility. The majority of these issues were tackled in the computer lab session however since the completion of this study we have moved to using a web-based environment (WebCT) for learning resources related to the prescribing course and have embedded all relevant RLOs within this environment thus making student access to these tools easier. In addition we utilise an IT expert to provide an introduction to using WebCT session on the first day, and to provide technical support throughout the course.

### Students' perceptions of the impact of RLO learning following completion of the course

Ten students (7 female and 3 male) agreed to complete a telephone interview about the pharmacology RLOs used in the prescribing course. These 10 students represented approximately 15% of the total students in cohorts 2 and 3 and included students with a range of biological science qualifications.

When asked to recall the RLOs that supported the course all interviewees remembered using these learning tools;

'*Recall vividly, really brilliant, absolutely brilliant' *Participant 83 (male)

'*I just thought they were very good' *Participant 44 (female)

All 10 interviewees valued the RLO approach to teaching pharmacology (question 5) and would like to see more developed (question 8) while 50% of the interviewees had continued to access the RLOs since completing the course (question 3).

Two themes were apparent from the interview data one being the usefulness of the multimedia and reusability of the RLOs in terms of promoting learning in general;

*'Quite useful for myself because I need a mental picture of what is going on to be able to understand it' *Participant 81 (female)

*'For me it was the visual aspect, actually seeing the concept visually was a huge bonus for me because it just made things click. We'd had key lectures and I'd read about things, but I think for me just to see how things worked visually was what I needed to put the whole picture together' *Participant 82 (male)

*'It probably helped with my exam. To pass the exam yes. It kind of changes the slant, sometimes reading a book is difficult and listening to a person is sometimes difficult, it kind of gives a third opportunity to take information in in a slightly different way' *Participant 43 (male)

*'Sometimes I found the application of the pharmacology in understanding I found quite difficult. The RLOs when I used them several times, it clarified a great deal of information for me.' *Participant 83 (male) 

and the value of RLOs in promoting pharmacology understanding;

*'In our group we found the pharmacology lectures pretty intense and that (the RLOs) did actually simplify it a lot, yes it was definitely really useful' *Participant 41 (female)

*'Invaluable. For me as a mental health nurse practitioner the whole concept of pharmacology, pharmacodynamics, pharmacokinetics was a new concept so I would have really liked some RLOs for each area' Participant 82 *(male)

*'Because you are able to go at your own pace because pharmacology is the toughest part of the course from what I found and talking to other people. Studying it in that sort of fashion enables you to go at your own pace and go over again and again if you need which I certainly did' *Participant 44 (female)

Students were also asked in the 12 month follow-up if they were confident in their understanding of the pharmacology of the drugs they were prescribing. Of the 9 prescribers who answered this question six said they were confident, with two of them attributing at least part of this confidence to the RLOs. Perhaps just as importantly, one prescriber interpreted this question to be a measure of 'safety of practice';

'*7/10, feel safe enough but wouldn't say I know everything there is to know' *Participant 44 (female)

The links between prescribing confidence, safety and pharmacology knowledge were also highlighted by students in the questionnaire;

'For me the underlying knowledge and understanding given by the pharmacology input is very important in giving me confidence to prescribe'

'The more I revise the subject [pharmacology] the more the concepts fit together. It has had a great impact on my clinical decision making........ I feel I have a much greater understanding which has made me a safer practitioner'

### Students' perceptions of the impact of RLO learning on clinical practice

Students were asked whether the RLOs had had an impact on their clinical practice, and if so to provide examples, as part of the questionnaire and again during the 12 month follow up. On telephone interview 3 of the 8 prescribers who answered this question agreed that their practice had changed as a result of the knowledge gained through use of the RLOs.

With respect to the application of pharmacology to clinical practice students from cohorts 2 and 3, who had access to pharmacology RLOs, suggested that they valued the use of these learning tools in terms of constructing pharmacological understanding and integrating this into practice;

*'Reinforced need to take drugs regularly due to my understanding of half-lives eg paracetamol' *Cohort 2

*'I feel the increase in RLOs will be very useful, I have used these a lot as part of my revision programme – as they help to visualise and interpret different concepts' *Cohort 2

*'I have been less impulsive in prescribing decisions, I am more likely to try alternative measures first, rather than just advising medication as I would have done in the past, particularly in the elderly' *Cohort 3

This suggests that the use of RLOs as a learning tool did impact on student understanding of pharmacological concepts enough for them to utilise this knowledge within their clinical practice.

It can be seen from Table 9 that the biology qualifications possessed by the interviewed students ranged from none to 'A' level and that those students who continued to access the RLOs post-qualification were at the lower end of these qualifications. The RLOs utilised in this study seemed to be of particular value in supporting students with less underlying understanding of biological concepts, although this is likely to be a function of the level at which these particular RLOs were pitched rather than a function of RLOs themselves.

**Table 9:** Summary of responses from students post-qualification.

Gender	Science education (excluding nurse training)	Perceived impact of RLOs on practice (question 4)	Use of RLOs after finishing the course (question 3)	Value of RLO approach to teaching pharmacology (question 5)	Development of further pharmacology RLOs (question 8)	Confidence in pharmacology understanding of drugs prescribing (question 10)
F	O Level	Yes	Yes	Yes	Yes	Yes
F	O level	No	Yes	Yes	Yes	Yes
F	None	Yes – but not just RLOs	Yes	Yes	Yes	Yes
F	A Level	No	No	Yes	Yes	Yes
M	A Level	No	No	Yes	Yes	Yes to a certain extent
F	O Level	Yes	No	Yes	Yes	7/10 feel safe enough
F	O Level	No	No	Yes	Yes	Yes
M	O Level	N/A	Yes	Yes	Yes	It's a learning curve
M	CSE Level	N/A	Yes	Yes	Yes	N/A
F	O Level	No	No	Yes	Yes	Yes

Summary of student responses to questions in telephone interview one year following qualification as a nurse prescriber (n = 10).

There are a number of limitations of this study one of which is the lack of reliability data in relation to the pharmacology questionnaire used. The questionnaire was only distributed to students on a single occasion so test-retest reliability was not possible. Similarly in order to keep the questionnaire at a manageable length for students who are already asked to complete both module and teaching evaluations, the questions were limited in number and a measure of internal consistency was not possible. A further limitation is the fact that the study was based in a single institution and as such we are only able to assess the value of this educational intervention in our own setting. It remains to be seen whether this educational intervention retains its value in other institutional settings where pharmacology may be provided by staff from different professional backgrounds. This issue is however countered to some degree by comments received through the on-line evaluation of these open access RLOs from students at other institutions nationally and internationally.

'*I wish there were more of these. A great way to bring pharmacology to LIFE'*

'Thank you for this excellent website! I am currently coming to the end of my second year in Nursing (mental health) at the University of Surrey'

'*I will be glad if you provide me other tutorials regarding pharmacology *(MPhilPharmacol)'

We have also received specific requests from a prescribing course in New Zealand and from a Health Trust in Wales to embed these RLOs into their courses.

In terms of nurse prescribing education a recent study by Latter and colleagues suggested that only 22% of prescribing students felt that the education programme met their needs completely, 84% identified pharmacology as the area which was most commonly studied during private study and 39% felt that pharmacology was an area of knowledge which was needed for practice but not adequately covered by the education programme [27]. This correlates well with a study of mental health nurse prescribing students which reported a lack of confidence in pharmacology knowledge to the extent that students felt it insufficient to support their prescribing practice [28]. Moreover a recent study of nurse prescribers' conducted in the West Midlands reported that 26% of nurses had not prescribed since qualifying [29] suggesting a possible lack of confidence in their prescribing knowledge. These data appear to be reflected across the country with not as many nurses implementing their prescribing qualification as anticipated [30]. Initially these data seem to be in direct contrast to the recent national study of independent extended supplementary nurse prescribers conducted by Courtenay and colleagues [23] which indicated that 89% of nurses were confident in their prescribing practice. Whilst this survey collated responses from 868 nurses it should be borne in mind that only 73% of the initial sample responded and of these 87% had prescribed as an extended nurse prescriber (allowing them to prescribe independently from the extended nurse prescribing formulary) and 35% as a supplementary prescriber (allowing them to prescribe anything from the British National Formulary within an agreed client-specific clinical management plan drawn up in conjunction with a doctor) since qualifying. There are undoubtedly a large number of issues involved in determining whether nurses actually prescribe following qualification with pharmacology confidence being only one of these. It does seem irrefutable; however, that pharmacology is an area where nurses feel they need significant support even following qualification [23,27,28].

Perhaps one of the most important issues to arise from this study then is the level of confidence that students have post-qualification and the relationship between this and the pharmacology RLOs. The ability of nurses to continue to freely access this growing collection of pharmacology RLOs even following qualification, may act to expand their pharmacological understanding in terms of continuing professional development and to maintain their confidence levels.

## Conclusion

The introduction of RLOs dealing with specific pharmacological concepts significantly improved nurse prescribing students perceived understanding of the majority of these concepts. All RLOs were evaluated extremely positively by the students and telephone interviews with a small sample of students suggested that the RLOs were continuing to be used to support learning post-qualification and that a number of students felt their prescribing confidence was due, at least in part, to these educational tools. Developing an understanding of pharmacological concepts will allow nurse prescribing students to apply these fundamental concepts to their specific areas of clinical practice. This in turn will hopefully lead to increased confidence in their prescribing abilities thus ensuring that patients have a better access to medicines without compromising safety. This increase in confidence is perhaps even more important following the recent changes in government policy to discontinue the extended formulary for nurses and allow them independent access to the BNF.

Data from other institutions internationally which plan to utilise these RLOs will also be extremely valuable in determining the global value of pharmacology RLOs in improving understanding among prescribing students.

## Competing interests

The author(s) declare that they have no competing interests.

## Authors' contributions

FBH and HJW conceived and designed the study and obtained the funding. JSL acquired the questionnaire data and evaluation of RLO data. HJW performed statistical analysis of the data. JSL drafted the manuscript. All authors have read and approved the final manuscript.

## Pre-publication history

The pre-publication history for this paper can be accessed here:


